# Supplementary material for: Bootstrap aggregation for model selection in the model-free formalism
Source: Magn Reson (Gott). 2021 May 5;2(1):251–64. doi: 10.5194/mr-2-251-2021 (PMC8372780; doi:10.5194/mr-2-251-2021)
Supplement: The supplement related to this article is available online at: https://doi.org/10.5194/mr-2-251-2021-supplement. [file mr-2-251-supplement.zip › mr-2-251-2021-supplement-title-page.pdf]

Supplement of Magn. Reson., 2, 251–264, 2021
https://doi.org/10.5194/mr-2-251-2021-supplement
© Author(s) 2021. CC BY 4.0 License.

Supplement of

Bootstrap aggregation for model selection in the model-free formalism

Timothy Crawley and Arthur G. Palmer III

Correspondence to: Arthur G. Palmer III (agp6@columbia.edu)

- mr-2-251-2021-supplement-title-page.pdf
- Crawley_Bootstrap_Aggregation.ipynb

The copyright of individual parts of the supplement might differ from the article licence.
